# Human umbilical cord mesenchymal stem cells (hUC-MSCs) alleviate paclitaxel-induced spermatogenesis defects and maintain male fertility

**DOI:** 10.1186/s40659-023-00459-w

**Published:** 2023-08-13

**Authors:** YuSheng Zhang, YaNan Liu, Zi Teng, ZeLin Wang, Peng Zhu, ZhiXin Wang, FuJun Liu, XueXia Liu

**Affiliations:** 1 School of Bioscience and Technology, Weifang Medical University, Weifang, China; 2 Shandong Stem Cell Engineering Technology Research Center, Affiliated Yantai Yuhuangding Hospital of Qingdao University, Yantai, China

**Keywords:** Stem cell, Spermatogenesis, Sperm quality, Male fertility, Paclitaxel

## Abstract

Chemotherapeutic drugs can cause reproductive damage by affecting sperm quality and other aspects of male fertility. Stem cells are thought to alleviate the damage caused by chemotherapy drugs and to play roles in reproductive protection and treatment. This study aimed to explore the effects of human umbilical cord mesenchymal stem cells (hUC-MSCs) on alleviating paclitaxel (PTX)-induced spermatogenesis and male fertility defects. An in vivo PTX-induced mice model was constructed to evaluate the reproductive toxicity and protective roles of hUC-MSCs in male fertility improvement. A 14 day PTX treatment regimen significantly attenuated mice spermatogenesis and sperm quality, including affecting spermatogenesis, reducing sperm counts, and decreasing sperm motility. hUC-MSCs treatment could significantly improve sperm functional indicators. Mating experiments with normal female mice and examination of embryo development at 7.5 days post-coitum (dpc) showed that hUC-MSCs restored male mouse fertility that was reduced by PTX. In IVF experiments, PTX impaired sperm fertility and blastocyst development, but hUC-MSCs treatment rescued these indicators. hUC-MSCs’ protective role was also displayed through the increased expression of the fertility-related proteins HSPA2 and HSPA4L in testes with decreased expression in the PTX-treated group. These changes might be related to the PTX-induced decreases in expression of the germ cell proliferation protein PCNA and the meiosis proteins SYCP3, MLH1, and STRA8, which were restored after hUC-MSCs treatment. In the PTX-treated group, the expression of testicular antioxidant proteins SIRT1, NRF2, CAT, SOD1, and PRDX6 was significantly decreased, but hUC-MSCs could maintain these expressions and reverse PTX-related increases in BAX/BCL2 ratios. hUC-MSCs may be a promising agent with antioxidant and anti-apoptosis characteristics that can maintain sperm quality following chemotherapy treatment.

**Supplementary Information:**

The online version contains supplementary material available at 10.1186/s40659-023-00459-w.

## Introduction

Mammalian spermatogenesis is a complex cellular development process that involves the precise regulation of germ and somatic cells. This sequential process of mitosis, meiosis, and spermiogenesis is synergistically regulated by numerous signaling molecules [1, 2]. Any harmful factor within these signaling molecules can result in adverse effects on male fertility. Many factors, including genetic, endocrinological, environmental, and drug-related influences, may contribute to male infertility. Paternal exposure to harmful environmental and medical factors may cause defects in spermatogenesis and sperm quality, both of which can contribute to infertility [3, 4]. Most chemotherapy drugs will affect male fertility, but adolescent adults may want to have children after treatment, meaning that strategies for fertility protection and potential evaluation are particularly necessary [5].

A growing number of studies have shown that chemotherapeutic drugs commonly induce reproductive injury and have particularly nefarious effects on spermatogenesis and male fertility [6, 7]. Paclitaxel (PTX) is a widely used anti-tumor drug that exerts its therapeutic effects through microtubule aggregation [8]. The side effects of PTX on male reproduction are also evident. PTX can cause abnormalities in spermatogenesis, including reduced sperm count and motility, which may be associated with germ cell apoptosis [9]. PTX-induced increase in reactive oxygen species (ROS) levels is one of the major causes of germ cell damage. We previously reported that PTX treatment induced obvious reproductive damage in male mice, including impaired germ cell proliferation and meiosis. These changes may result in reduced sperm quality, including decreased sperm counts and motility, as well as higher proportions of malformed sperm, which together lead to reduced male fertility [9].

The physiological levels of ROS help regulate normal spermatogenesis processes, while disruptions in the oxidant-antioxidant system can retard testicular growth and disrupt spermatogenesis, leading to male infertility [10]. Antioxidants such as melatonin may partially alleviate PTX-induced oxidative damage, thus protecting sperm quality [9]. In addition to antioxidants and other exogenous drugs, the application of MSCs in reproductive therapy has become a popular area of research. MSCs can actively differentiate and self-renew. They play an important role in repairing cell damage, resistance to cellular aging, and anti-inflammatory, and anti-oxidative damage processes [11]. MSCs derived from umbilical cords (UC-MSCs), bone marrow (BM-MSCs), and human amnion membranes (hA-MSCs) can significantly improve busulfan-induced testicular damage [12–14]. BM-MSCs have been shown to protect rat testes via anti-inflammatory and immune-modulatory pathways as well as modulatory effects on oxidative stress [15]. hA-MSCs could improve ionized radiation-induced testicular damage by reducing ER stress and apoptosis [16]. These studies provide important background information for further investigation of the application of MSCs in male reproductive health.

Here, we investigated the protective role of hUC-MSCs against PTX-induced reproductive damage in males by examining the spermatogenesis processes of germ cell proliferation and meiosis, and sperm fertility potential in vivo and in vitro. hUC-MSCs could significantly resist the oxidative damage caused by PTX, promote germ cell proliferation, maintain sperm quality, and restore defects in sperm fertility induced by PTX. These findings provide valuable clues for further research about MSCs in male reproduction.

## Materials and methods

### Isolation and culture of hUC-MSC

hUC-MSCs were cultured and identified as reported [17]. Briefly, the umbilical cord was obtained from pregnant women giving birth, who gave informed consent for umbilical cord collection. The procedure and subsequent use of the umbilical cord were approved by the ethical review committee of Yantai Yuhuangding Hospital (Approval NO. 2021-118, 2021-11-28). The Wharton's jelly was isolated from the umbilical cord and attached to culture plates supplemented with minimum essential medium α (MEMα) (C3060–0500, Biological Industries, Kibbutz, Israel) containing 10% fetal bovine serum (FBS) (C04001–500, Biological Industries, Kibbutz, Israel). After the colonies appeared to have 80% confluence, the cells were cultured into new plates. The cells cultured at passages 3–5 were used for the present study.

### Cell identification for hUC-MSC

hUC-MSCs were characterized by the expression of cell surface markers. Briefly, the fourth passage cells at a concentration of approximately 5 × 10^6^ were harvested, and resuspended at a concentration of 10^5^ cells/500 μl in phosphate buffered solution (PBS) and incubated with monoclonal antibodies labelled with different fluorophores: CD34 (APC), CD45 (FITC), CD19 (PE), CD11b (R718), HLA-DR (RB780), CD44 (FITC), CD73 (BV510), CD105 (APC), and CD90 (FITC) and corresponding isotype controls (BD Biosciences Pharmingen, San Diego, CA, USA), respectively. After incubation for 30 min at room temperature (RT), hUC-MSCs were washed three times and resuspended in 500 μl PBS for flow cytometry analysis (MoFlo XDP, Beckman, USA). Cell death rates were analyzed using Trypan blue (C0011, Beyotime, Shanghai, China) staining.

In regard to multilineage differentiation, hUC-MSCs of passage 4 were cultured at a concentration of 3 × 10^4^/well in a 6-well plate. After 80–90% confluence, MSCgo^™^ Osteogenic Differentiation Medium and MSCgo^™^ Adipogenic Differentiation Medium (05-440-1B, 05-330-1, Biological Industries, Kibbutz, Israel) were used to induce osteogenesis and adipogenesis, respectively. As for adipogenic differentiation, after differentiation culturing for 14–21 days, the medium was changed into MSC NutriStem XF Medium (05-200-1, Biological Industries, Kibbutz, Israel) for 3–4 days until lipid droplets formation. Alizarin Red S and Oil red-O were used to detect of successful osteogenic and adipogenic differentiations, respectively. In addition, 1 × 10^5^/10 μl cells at the fourth passage were cultured in a U-bottom 96-well plate for 2 h to promote pellet formation, then 100 μl MSC NutriStem XF Medium was added into 96-well for 24 h. After 24 h, MSCgo^™^ Chondrogenic Differentiation Medium (05-220-1B, Biological Industries, Kibbutz, Israel) was used to replace the above medium for subsequent 14–21 days culture. The pellet was fixed in 4% formaldehyde, embedded in paraffin, cut into 4-μm-thick sections, and stained with Alcian Blue to detect chondrogenic differentiation.

### Animal experiments

The Institute of Cancer Research (ICR) mice (6–8 weeks old, 30–35 g) were purchased from Beijing Vital River Laboratory Animal Technology Company. The mice were kept at a constant temperature (24 ± 2 ℃) and 12/12 light–dark cycle. The animals were free of food and drinking water. The experimental protocol was approved by the Ethical Committee on Animal Research of Yantai Yuhuangding Hospital.

As for the preliminary experiment, the mice were divided into 6 groups randomly as follows: (1) Control group: animals were injected physiological saline solution intraperitoneally followed by PBS (200 μl/mouse) 1 day later via tail vein (n = 3); (2) PTX group: animals were injected intraperitoneally with PTX solution (5 mg/kg body weight) once followed by PBS (200 μl/mouse) 1 day later via tail vein (n = 3); (3–6) PTX + hUC-MSCs groups: animals were injected intraperitoneally with PTX solution followed by different concentration of hUC-MSCs (5 × 10^5^/200 μl/mouse, 1 × 10^6^/200 μl/mouse, 2 × 10^6^/200 μl/mouse, and 5 × 10^6^/200 μl/mouse, n = 3, respectively) 1 day later via tail vein. A week later, mice were euthanized (1.25% 2,2,2-Tribromoethanol sterile anesthetic, 0.2 ml/kg, intraperitoneal injection). The testes were collected for morphology examination and antioxidant capacity detection.

For further investigation, the mice were divided into 3 groups randomly as follows: (1) Control group: animals were injected with physiological saline solution intraperitoneally once followed by PBS (200 μl/mouse) 1 day later via tail vein (n = 10); (2) PTX group: animals were injected intraperitoneally with PTX solution (5 mg/kg body weight) once followed by PBS (200 μl/mouse) 1 day later via tail vein (n = 10); (3) PTX + hUC-MSCs group: animals were injected intraperitoneally with PTX solution followed by hUC-MSCs (2 × 10^6^/200 μl/mouse, based on preliminary experiment results) 1 day later via tail vein (n = 10). Two weeks later, mice were euthanized for subsequent experiments.

Normal estrous female mice were selected and mated with male mice in different groups at a ratio of 3:1. In the following morning, female mice with vaginal plug were identified for further observation to determine pregnancy status. To assess the embryo development, vaginal plug formation was recorded as 0.5 post-coitum (dpc), while pregnant female mice were euthanized at 7.5 dpc under anesthesia. Male fertility was evaluated by calculating the ratio of pregnant female mice to the number of female mice with vaginal plugs.

For external superovulation, normal female mice were injected intraperitoneally with 10 i.u. pregnant mare serum gonadotrophin (PMSG) and human chorionic gonadotrophin (hCG) 48 h apart. Each female mouse could produce about 35 oocytes, which were collected and cultured in a HTF medium. Mice cauda epididymal sperm in each group were collected for sperm capacitation in 200 μl CTYH medium for 0.5 h. About 10^6^ sperm/ml was transferred into a human tubal fluid (HTF) medium containing oocytes. All procedures were done in a humidified incubator at 37 ℃ with 5% CO_2_. After sperm-egg incubation for 4–6 h, the developed embryos were recorded, and the fertility rate was defined as a ratio of pronuclear formation embryos/obtained oocytes. Pronuclear formation embryos were transferred into a balanced KSOM medium with a paraffin overlay for the following culture. The percentage of the two-cell embryos/the number of pronucleus formation oocytes was regarded as embryo development rate and the blastocytes/the number of the two-cell embryos was recorded as blastocyst rate.

### Histological assay

After fixation in Bouin's solution for 12 h, mice testes were embedded in paraffin. The slides with 4 μm thickness were prepared. After dewaxing and dehydration, the slides were stained with hematoxylin and eosin (HE), and the morphological structures were observed under a light microscope (DM LB2, Leica, Germany).

### TUNEL assay

TUNEL analysis was performed according to manufacturer’s instruction (BA2520, Biobox, Nanjing, China). The sections were de-waxed and dehydrated in xylene and ethanol, followed by incubation with proteinase K for 30 min at 37 ℃. After being immersed in blocking solution (0.1 M Tris–HCL PH7.5, 3% BSA, AND 20% FBS) for 10 min at RT, the sections were stained with a TUNEL detection solution. Images were captured by fluorescent microscopy (Observer 7, Carl Zeiss, Jena, Germany).

### Immunohistochemical and immunofluorescent staining

Immunohistochemistry and immunofluorescent analysis were performed as previously described [18]. Briefly, the sections were dewaxed and dehydrated in xylene and ethanol, respectively. Then the sections were reacted with 3% H_2_O_2_ at RT for 5 min to remove endogenous hydrogen peroxides, and incubated with 3% bovine serum albumin (BSA) at RT for 1 h to block non-specific bindings. The sections were then incubated with primary antibody at 4 ℃ overnight, and following incubated with secondary antibody at RT for 1 h. 3, 3′- diaminobenzidine (DAB) Kit (ZLI-9018, Zhong-Shan Golden Bridge, Beijing, China) was finally applied to display the peroxidase activity at the binding sites. Harris hematoxylin solution (ZLI-9609, Zhong-Shan Golden Bridge, Beijing, China) was used to stain nuclei. The stained sections were dehydrated and observed under a light microscope (DM LB2, Leica). For immunofluorescent staining, the slides were incubated with corresponding fluorescent secondary antibody and PI or DAPI (1 μg/ml) (P0135, P0131, Beyotime, Shanghai, China) was used to stain nuclei. Fluorescent images were captured with a fluorescent microscope (Observer 7, Carl Zeiss, Jena, Germany) and analyzed by ImageJ software. The primary antibodies included Nuclear factor-erythroid 2-related factor 2 (NRF2) (AF7006, Affinity, Biosciences, JiangSu, China), Heat Shock 70 kDa Protein 2 (HSPA2) (ab108416), HSPA4L (ab231577), Proliferating cell nuclear antigen (PCNA) (ab92552), Sirtuin 1 (SIRT1) (ab189494, Abcam, Cambridge, UK), STEM121 (Y40410, Cellartis, Takara, Japan), and CD73 (AG2762, Beyotime, Shanghai, China).

### *Tracking of hUC-MSCs *in vivo* after injection*

hUC-MSCs (1 × 10^8^) were labelled with 5(6)-Carboxyfluorescein diacetate succinimidyl ester (CFDA-SE) (C0051, Beyotime, Shanghai, China). The labeled cells were washed three times with PBS and injected into 24 mice via tail vein 24 h after PTX treatment. The distribution of hUC-MSCs in the mice heart, liver, spleen, lung, kidney, and testis was detected at 8 different time points within 72 h (15 min, 30 min, 3 h, 6 h, 12 h, 24 h, 48 h and 72 h). Fluorescence signals in frozen sections (5 μm) were detected by a fluorescence microscope (Observer 7, Carl Zeiss, Jena, Germany) at a magnification of × 20.

### Oxidative stress indicators detection

Oxidative stress related markers of superoxide dismutase (SOD) (S0103, Beyotime, Shanghai, China) and glutathione (GSH) (S0052, Beyotime, Shanghai, China) were detected. The testis tissue or cell lysate was extracted and measured according to manufacturer’s instruction. Optical density (OD) values were read using microplate reader (Varioskan, Thermo Scientific, Shanghai, China) and analyzed by ImageJ software.

### Western blotting

Protein extractions were performed by grinding the tissues in liquid nitrogen, and lysing in Radio Immunoprecipitation Assay (RIPA) buffer (P0013, Beyotime, Shanghai, China) containing protease and phosphatase inhibitor cocktail (P1011, Beyotime, Shanghai, China). The protein concentrations were measured by a BCA protein assay kit (P0012, Beyotime, Shanghai, China). Equal proteins (50 μg/lane) from each sample were separated by 12% SDS-PAGE gels, transferred onto a nitrocellulose filter membrane and blocked with 5% (w/v) skimmed milk in tris-buffered saline containing 0.1% Tween 20 (TBST). The membranes were then hybridized overnight at 4 ℃ with primary antibodies: Proliferating cell nuclear antigen (PCNA), ab92552; Synaptonemal complex protein 3 (SYCP3), ab97672, Bcl-2-associated X protein (BAX), ab32503; B-cell lymphoma 2 (BCL2), ab182858; β-Tubulin (TUBB), ab179513, Abcam, Cambridge, UK; MutL homolog 1 (MLH1), D221003; DNA meiotic recombinase 1 (DMC1), D224646; Meiotic recombination protein (REC8), D222997; Catalase (CAT), D222036; Superoxide dismutase 1 (SOD1), D221245; Peroxiredoxin 6 (PRDX6), D121159, BBI, Shanghai, China). After washing with TBST for 3 times, the membrane was incubated with appropriate HRP-conjugated secondary antibody (ZB2305, Zhong-Shan Golden Bridge, Beijing, China, 1:5000) at RT for 1 h. After being washed by TBST, the protein bands were detected by an ECL kit (KF8001, Affinity Biosciences, JiangSu, China) using ChemiScope 6200 Touch (CLINX Science Instruments Co.,Ltd. China) and quantified with ImageJ software using TUBB as the loading control.

### RNA isolation and real-time quantitative PCR

Total RNA was extracted from mice testis with RNA isolated Total RNA Extraction Reagent (R701-01, Vazyme, Nanjing, China) following the manufacturer’s instructions. 5 × All-In-One RT MasterMix with AccuRT (G592, Abm, Jiangsu, China) was used to synthesize cDNA from total RNA. RT-qPCR was performed with BlasTaq 2 × qPCR MasterMix (G891, Abm, Jiangsu, China) on ABI Prism 7500 (Thermo Scientific, Shanghai, China). The comparative delta cycle threshold (CT) method was used to calculate the relative expression levels of each target gene to *Actb*. All experiments were conducted 3 times. The primer sequences were as follows:

*mSod1*-F: AACCAGTTGTGTTGTCAGGAC, *mSod1*-R: CCACCATGTTTCTTAGAGTGAGG; *mCat*-F: TGGCACACTTTGACAGAGAGC, *mCat*-R: CCTTTGCCTTGGAGTATCTGG; *mPrdx6*-F: CATCCTTTTGGGCATGTTGG, *mPrdx6*-R: TGGCAGGGTAGAGGATAGAC; *mActb*-F: GCAGCTCAGTAACAGTCCGC, *mActb*-R: AGTGTGACGTTGACATCCGT.

*Actb* served as the control gene. The CT of each gene was recorded. △CT was the difference value of CT_*Gene*_ -CT_*Actb.*_ △△CT of each gene was the difference value of △CT_treatment_ -△CT_control_. The results were displayed as 2^−△△CT^, and statistically analyzed by One-Way ANOVA.

### Statistical analysis

All data were analyzed as the mean ± standard deviation of three independent repeats. The data were performed on normality tests, which used Anderson–Darling test. Then One-Way ANOVA analysis was performed by using Tukey test for multiple comparisons. GraphPad Prism 9 (GraphPad Prism, La Jolla, CA) was used to perform all the analysis, a *p* value less than 0.05 was considered significant.

## Results

### Characterization of hUC-MSCs

**Fig. 1:**
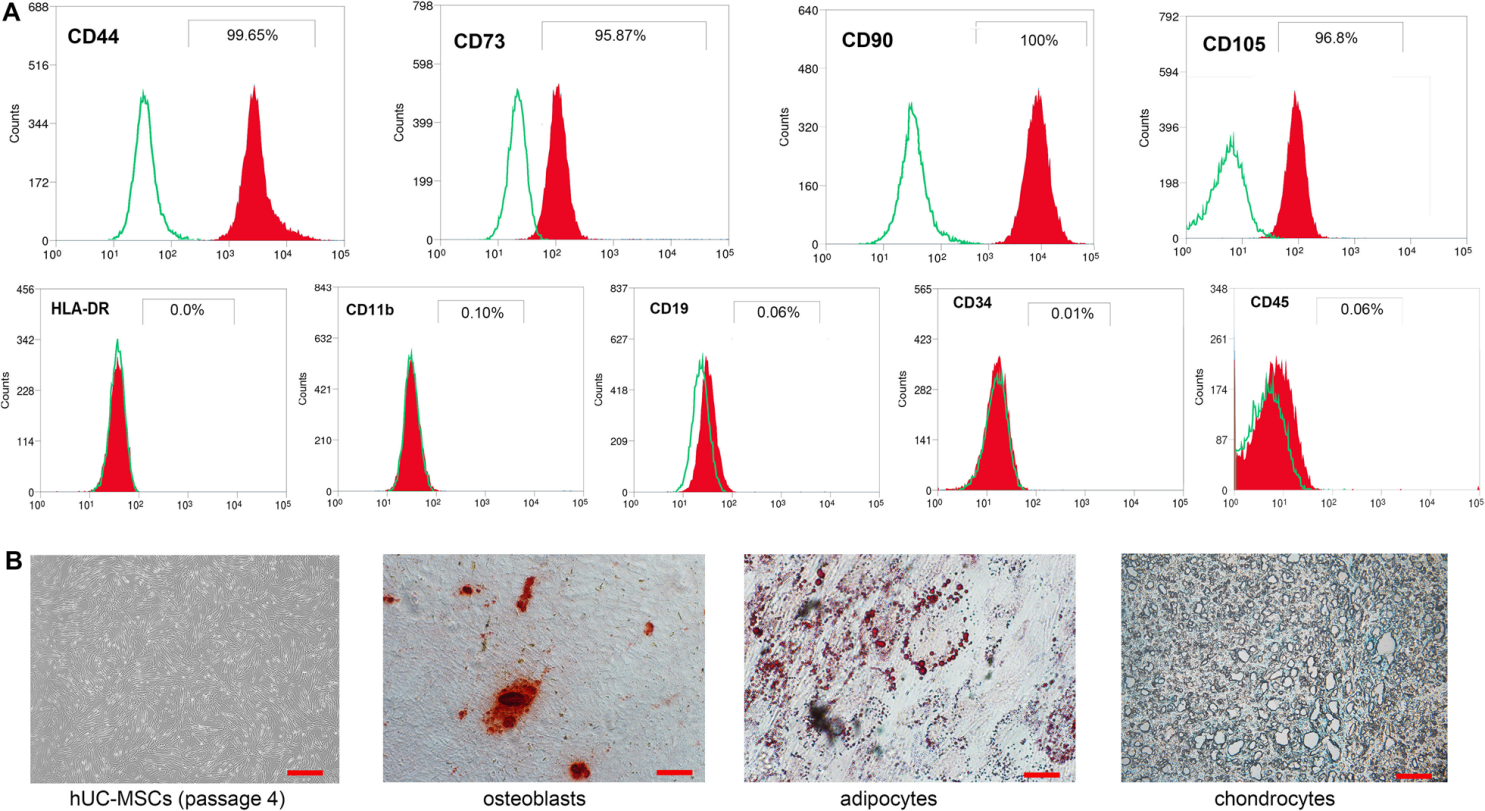
Characteristics identification of human umbilical cord mesenchymal stem cells (hUC-MSCs) **A**: CD44, CD73, CD90 and CD105 exhibited positive expressions in hUC-MSCs, and CD19, CD34, CD45, CD11b, and HLA-DR exhibited negative expressions. **B**: Fibroblast-liked hUC-MSCs differentiated into osteoblast, chondrocyte and adipocyte in vitro. Each bar represented 20 μm

The hUC-MSCs used in this study were verified to show fibroblast-liked morphology, and could differentiate into osteoblast, chondrocyte and adipocyte in vitro. Flow cytometric analysis confirmed that these cells expressed CD44, CD73, CD90 and CD105 (positive cells ≥ 95%), but not CD11b, CD19, CD34, CD45 and HLA-DR (positive cells ≤ 2%). The average cell purity was 98% (Fig. 1). Thus, these hUC-MSCs met the criteria of the guidelines from the Mesenchymal and Tissue Stem Cell Committee of the International Society for Cellular Therapy (ISCT) [19].

**Fig. 2:**
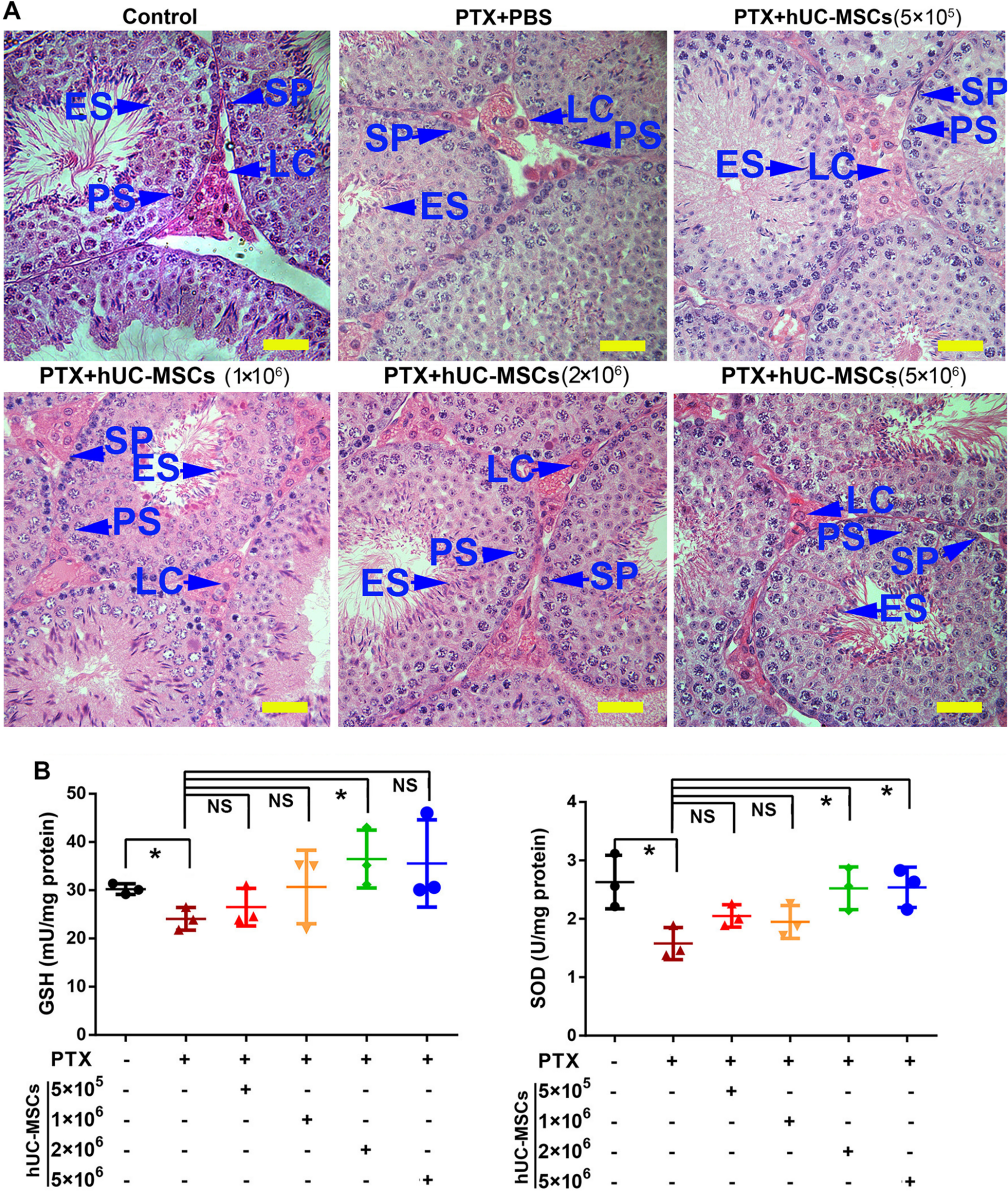
Morphological analysis and detection of GSH and SOD levels in the testes of mice treated with different concentrations of hUC-MSCs The mice were treated with PTX or hUC-MSCs, and samples were collected 1 week after the treatment. **A**: The representative images were stained by HE; **B**: GSH and SOD levels were detected in testicular homogenate; PTX, Paclitaxel; hUC-MSCs, human umbilical cord mesenchymal stem cells; The data were analyzed by one-way ANOVA; *p* value less than 0.05 was considered significance; *, *p* < 0.05; **, *p* < 0.01; ***, *p* < 0.001; Each bar represented 20 μm. *SP* Spermatogonia, *PS* Pachytene spermatocytes, *RS* Round spermatids, *ES* Elongated spermatids, *LC* leydig cells

To select the appropriate hUC-MSCs concentration for the treatment of PTX-induced mice, we compared the testicular morphology alterations and antioxidant capacity after 1 week treatment with different dosages of hUC-MSCs. The results showed that PTX induced mice testis morphology changes including decreased height of germ cell layers, reduced germ cell numbers and interstitial hyperemia. PTX also induced significantly decreased GSH and SOD levels in the testis. hUC-MSCs treatment alleviated the PTX-induced damages in the testis, and 2 × 10^6^ cells and 5 × 10^6^ cells hUC-MSCs yielded better therapeutic results (Fig. 2, Additional file 1: Fig. S1). Thus, hUC-MSCs with a moderate dosage of 2 × 10^6^ cells were selected for further investigation.

**Fig. 3:**
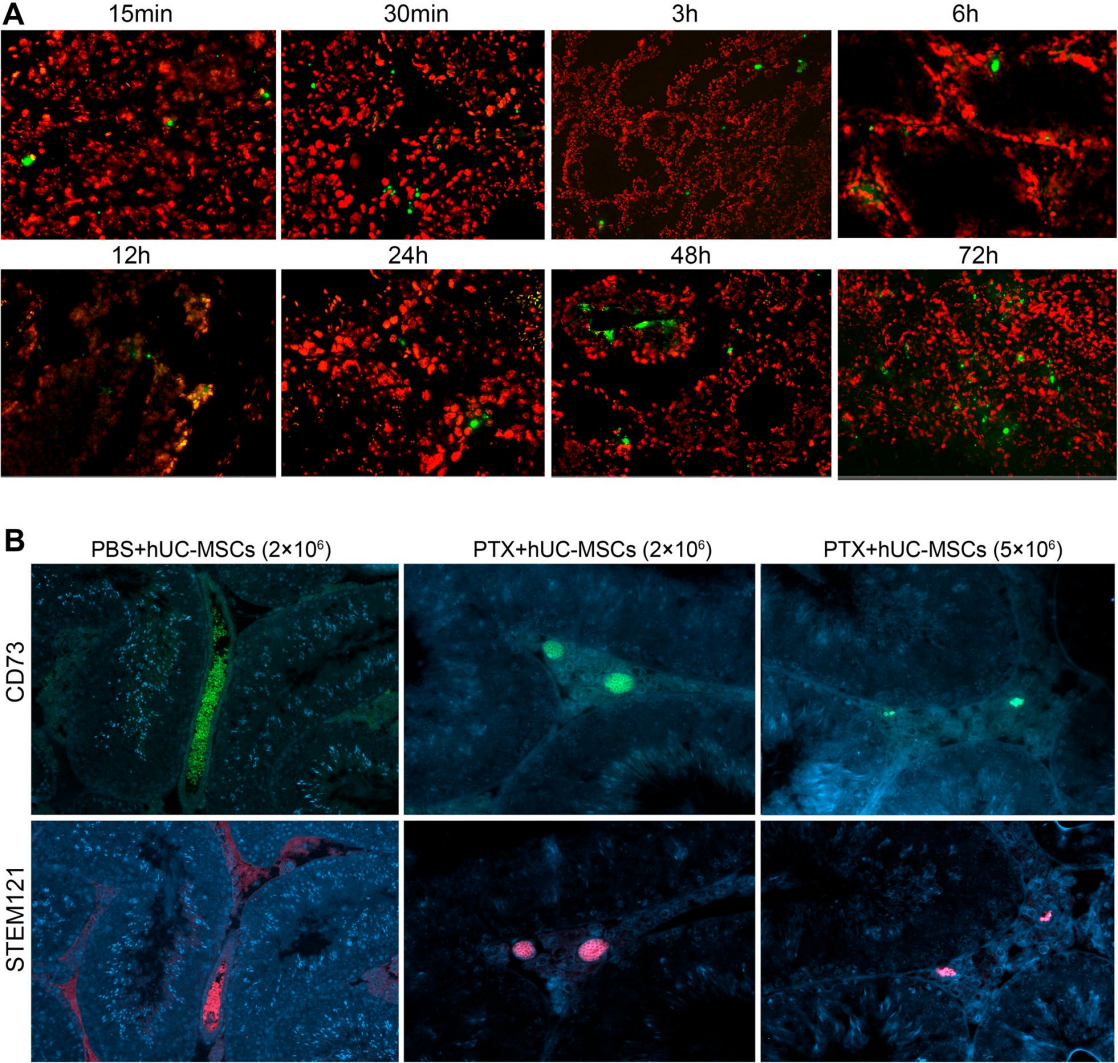
Tracking of labelled hUC-MSCs in mice testis **A**: Detection of CFDA-SE labelled hUC-MSCs under fluorescence microscope in mice testis at different time points after injection. The green signals in the frozen section represent the labeled hUC-MSCs; **B**: Detection of CD73 and STEM121 in mice testis with positive hUC-MSCs 1 week after the injection. Green signals indicated the CD73-stained hUC-MSCs, red signals indicated STEM121-stained hUC-MSCs and blue signals indicated DAPI-stained nuclei

CFDA-SE labeled hUC-MSCs were used to track the distribution of cells after injection. Fluorescence signals began to accumulate in the spleen 15 min after injection, and were gradually detected in the heart, kidney, lung and liver (Additional file 2: Fig. S2). In mice testis, signals appeared at 15 min after injection (Fig. 3A). CD73 and STEM121 were used for better tracking the cell distribution. We detected the expression of CD73 and STEM121 in control (PBS + hUC-MSCs) and PTX + hUC-MSCs treated mice testis 1 week later. CD73 and STEM121-positive signals were clearly present in interstitial vessels (Fig. 3B).

### hUC-MSCs improved PTX-induced decreases in sperm quality in mice

**Fig. 4:**
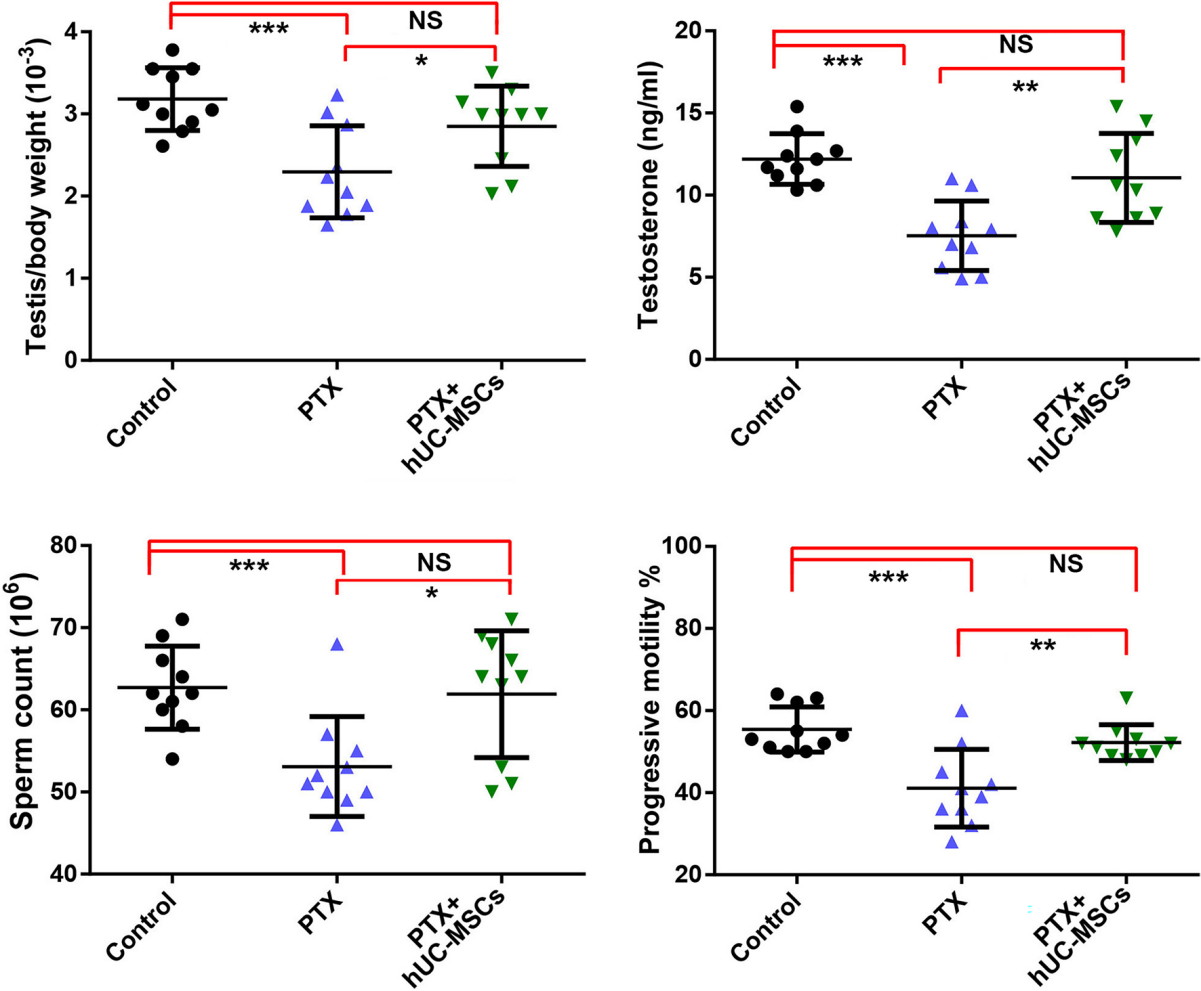
Characteristics of testis/body weight, testosterone concentration, sperm counts and sperm motility in PTX-treated and hUC-MSCs-treated mice. The mice were treated with PTX or hUC-MSCs, and samples were collected 2 weeks later. Data of each group were obtained from ten mice and analyzed by one-was ANOVA; *p* value less that 0.05 was significant; *, *p* < 0.05; **, *p* < 0.01; ***, *p* < 0.001

Our previous study indicated that the PTX induced changes in spermatogenesis and sperm quality could be detected at the molecular level from day 14 onwards [9]. Therefore, we selected the PTX-treated mice at 14 days in order to investigate the protective roles of hUC-MSCs. Consistent with our previous study, PTX treatment significantly decreased testis weight, serum testosterone levels, sperm counts, and progressive sperm motility, suggesting poor sperm quality in PTX-treated mice. Sperm counts and sperm motility represent the fertility capabilities of sperm, while serum testosterone is critical for spermatogenesis and sperm quality. Administration of hUC-MSCs significantly improved the above indicators associated with sperm quality (Fig. 4). These results suggested that MSC significantly recovered spermatogenesis changes induced by PTX treatment.

**Fig. 5:**
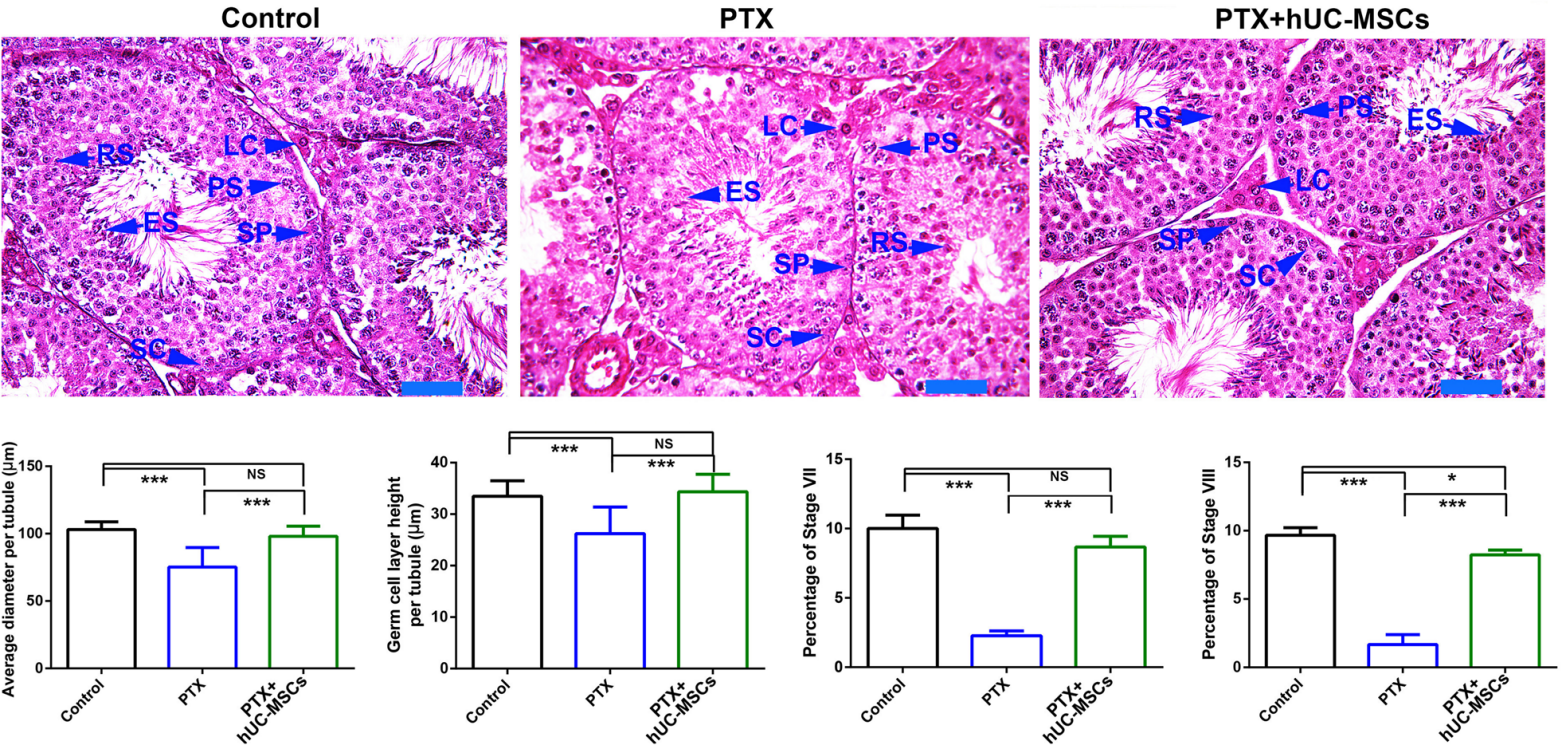
Morphological analysis of testes from control, PTX-treated and PTX + hUC-MSCs treated mice. The mice were treated with PTX or hUC-MSCs, and samples were collected 2 weeks later. he sections were demonstrated by HE staining. The data were analyzed by one-way ANOVA; * represented *p* value less than 0.05; *, *p* < 0.05; **, *p* < 0.01; ***, *p* < 0.001; Each bar represented 20 μm

Morphological examination of testicular seminiferous tubules by HE staining demonstrated that hUC-MSCs could reduce the cell damages caused by PTX, including reduced numbers of germ cells within tubules and loose interstitial structures. hUC-MSCs could help maintain normal percentages of stage VII and VIII seminiferous tubules, which were significantly reduced in PTX-treated mice (Fig. 5).

### Male fertility was maintained in PTX+hUC-MSCs treated mice

**Fig. 6:**
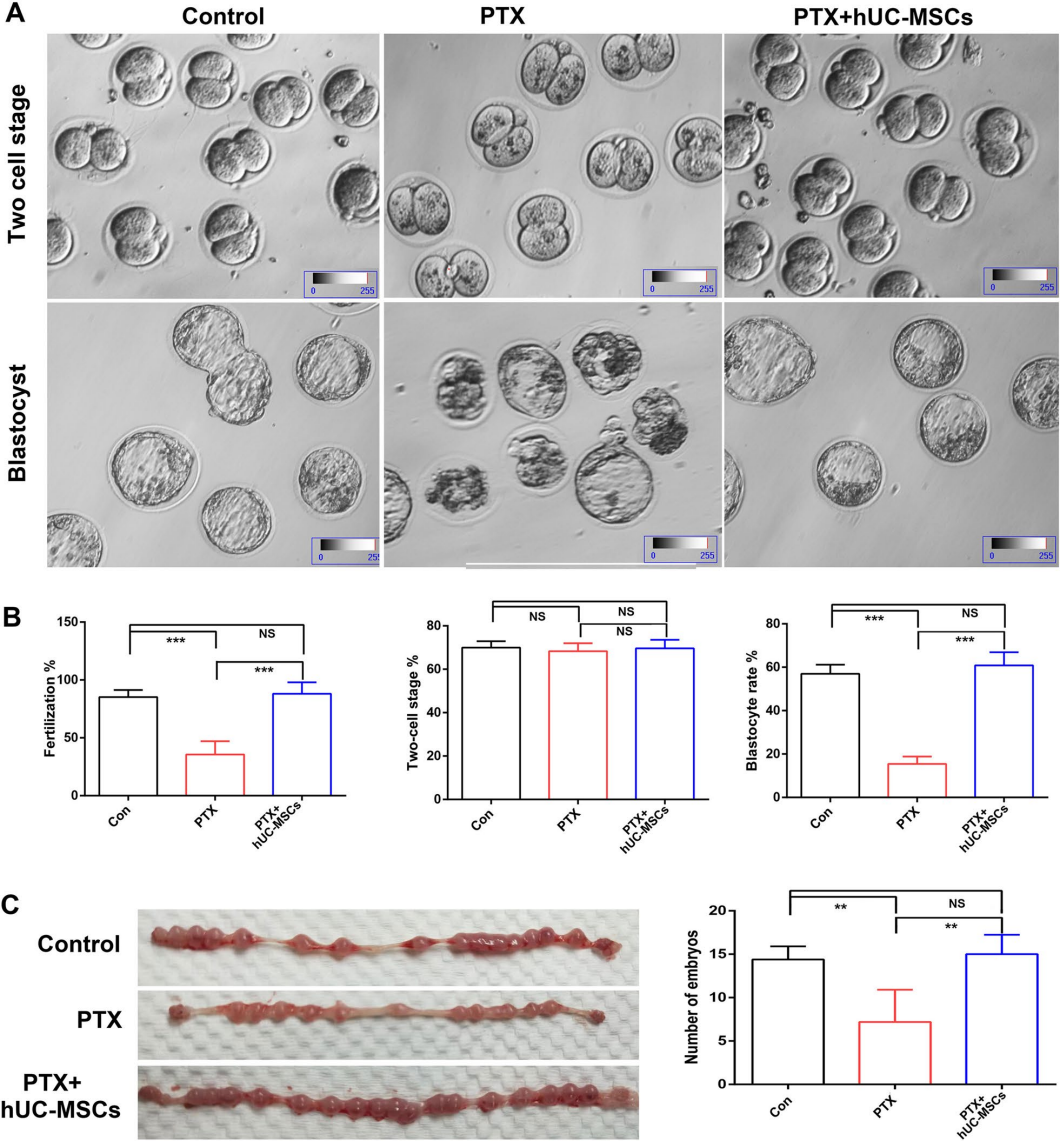
In vitro embryo development analysis. Cauda epididymal sperm from different groups were used for IVF analysis; Oocytes were obtained from normal female mice; **A**: embryo development in different group by IVF; **B**: Statistical analysis of the rate of fertilization, two-cell and blastocyst development in different group by one-way ANOVA; **C**: Statistical analysis of the number of embryos at 7.5 dpc by one-way ANOVA; *p* value less than 0.05 was considered significance; *, *p* < 0.05; **, *p* < 0.01; ***, *p* < 0.001

Male fertility ability was assessed using in vivo and in vitro analysis. After mating with normal female mice, PTX-treated mice led to decreased female mice pregnancy rates and litter numbers compared to those in the control and hUC-MSCs treatment groups (Table 1). This male fertility defect might be attributable to the poor sperm quality induced by PTX. We subsequently verified sperm fertility through in vitro experiments and found that PTX significantly reduced rates of sperm fertility and blastocyst formation but not two-cell embryo development. hUC-MSCs rescued the decreased sperm fertility and blastocyst formation ratios. We measured embryonic development 7.5 days after vaginal plug examination and found that PTX treatment resulted in decreased embryo formation, while hUC-MSCs significantly neutralized the detrimental effects of PTX (Fig. 6).

**Table 1:** Fertility and fecundity of different treatment mice

Group	Male fertility	Litter numbers
Control	93.33% (14/15)	9.00 ± 0.93
PTX	73.33% (11/15)^a^	7.72 ± 1.21^a^
MSCs + PTX	100% (15/15)	8.80 ± 0.83

Mice of the indicated groups were caged with normal female mice. Male fertility were shown as the number of pregnant female mice/the number of female mice with vaginal plugs. The litter numbers were the average number of pups born from pregnant females

^a^Represented *p* value less than 0.01

### hUC-MSCs maintained germ cell proliferation and meiosis

**Fig. 7:**
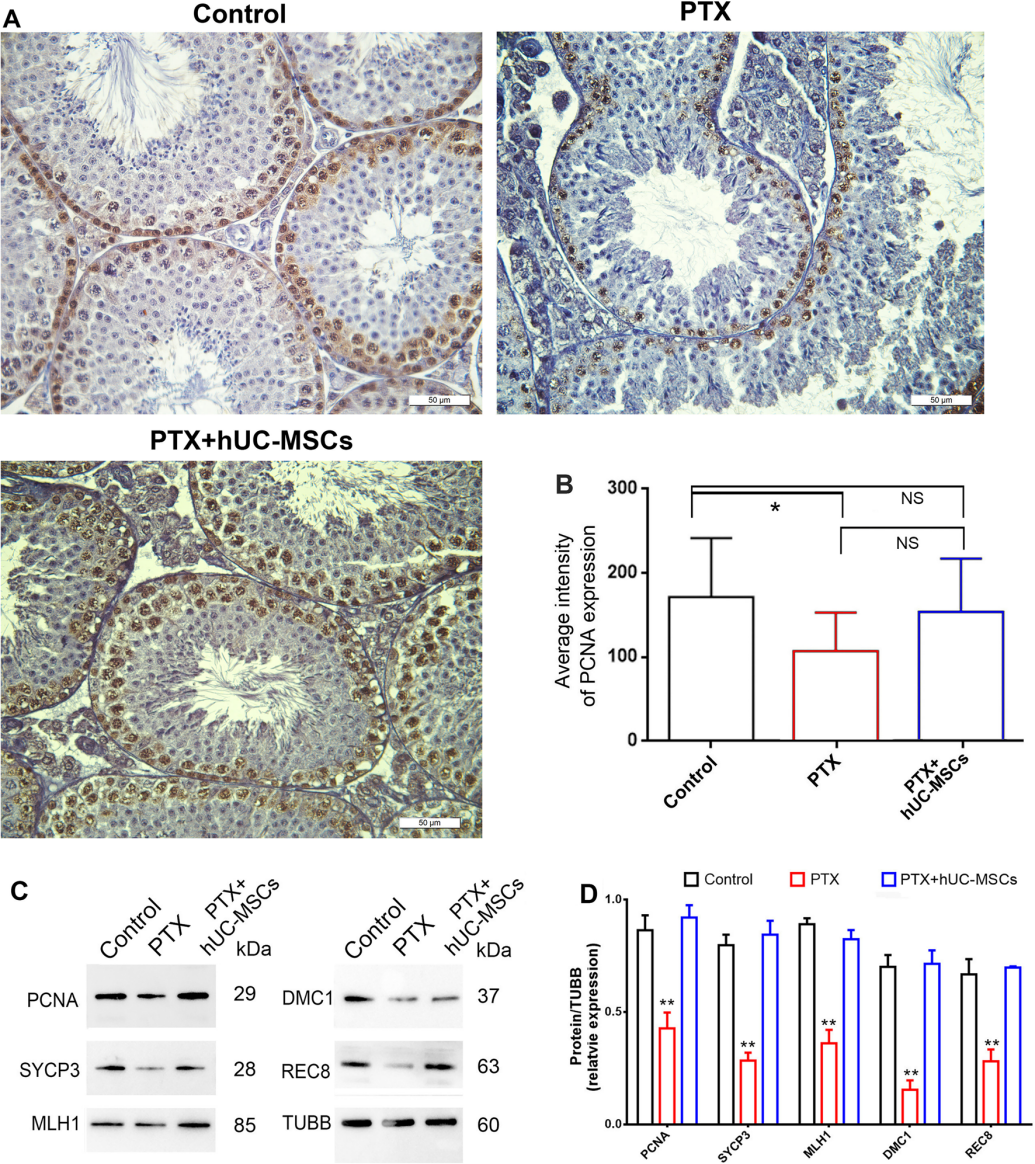
Expressions of PCNA and meiosis related proteins in control, PTX treatment and PTX + hUC-MSCs treatment mice testis. The mice were treated with PTX or hUC-MSCs, and samples were collected 2 weeks later. **A**, **B**: Testicular expression of PCNA in mice of control, PTX and PTX + hUC-MSCs group; **C**, **D**: Expressions of PCNA, SYCP3, MLH1, DMC1, and REC8 in mice testes of control, PTX and PTX + hUC-MSCs group; Statistical analysis was performed by One-Way ANOVA; The arrow indicated the positive staining;* p* value less than 0.05 was considered significance; *, *p* < 0.05; **, *p* < 0.01; ***, *p* < 0.001; Each bar in (**A**) represented 50 μm

We previously found that PTX treatment mainly affected germ cell proliferation and meiosis by reducing key protein expression in testicular tissue. Here, we detected the expression of PCNA (proliferation-related protein) and SYCP3, MLH1, DMC1, and REC8 (meiosis-associated proteins) in germ cells using immunohistochemistry and Western blotting. The results showed decreased expression of these proteins in PTX-treated mice compared with control mice, but hUC-MSCs could reverse the declines and even restore normal levels (Fig. 7).

### hUC-MSCs improved the expression of fertility-related proteins HSPA2 and HSPA4L

**Fig. 8:**
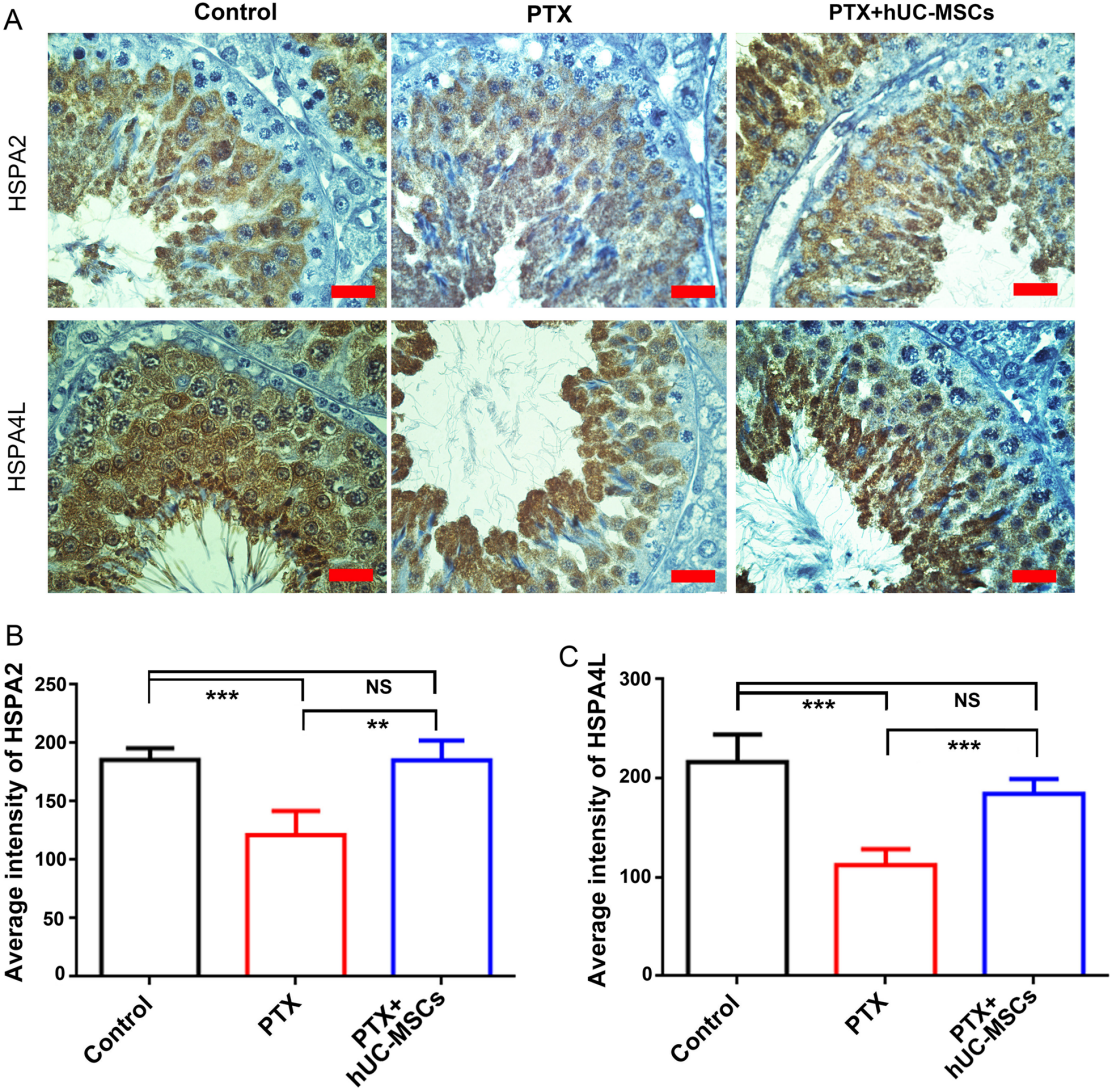
The expressions of fertility protein HSPA2 and HSPA4L in control, PTX treatment and PTX + hUC-MSCs treatment group. The mice were treated with PTX or hUC-MSCs, and samples were collected 2 weeks later. **A**: The cellular expressions of HSPA2 and HSPA4L in testes of different groups; The statistical analysis of average intensity of positive staining of HSPA2 (**B**) and HSPA4L (**C**) in different groups; *, *p* < 0.05; **, *p* < 0.01; ***, *p* < 0.001; Each bar in (**A**) represented 20 μm

The fertility-related proteins HSPA2 and HSPA4L are highly abundant in the testis. Their expressions in PTX and PTX + hUC-MSCs mice were detected by immunohistochemistry. HSPA2 and HSPA4L were mainly expressed in germ cells. HSPA2 was mainly expressed in spermatocyte cells as well as round and elongated spermatids, and HSPA4L was expressed in all germ cells. Statistical analysis demonstrated that the average positive staining intensities of HSPA2 and HSPA4L were significantly reduced in the PTX group, while hUC-MSCs ameliorated these decreases in expression (Fig. 8).

### hUC-MSCs attenuated oxidative stress caused by PTX

**Fig. 9:**
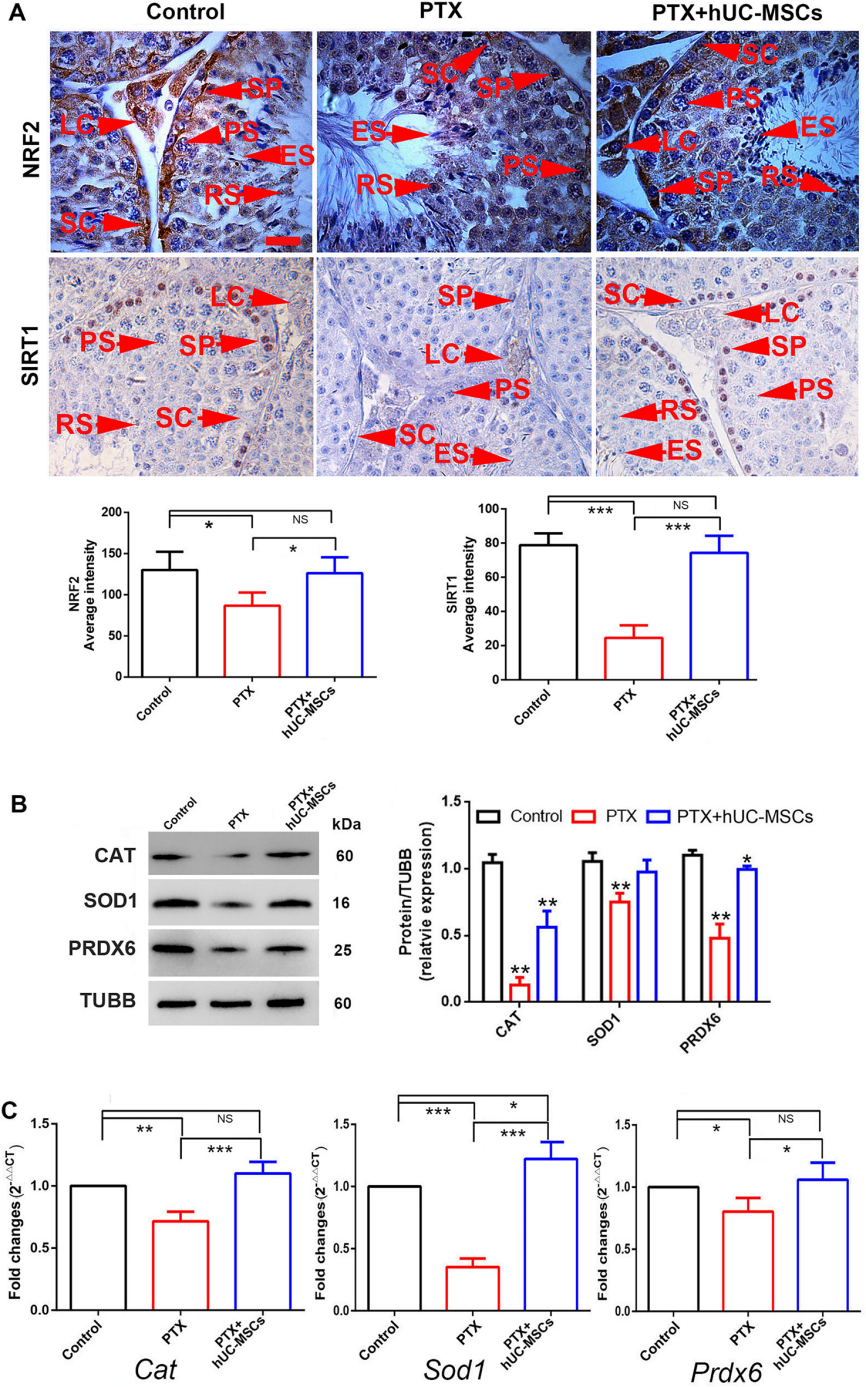
Expressions of NRF2, SIRT1 and antioxidant molecules of CAT, SOD1, PRDX6 in control, PTX treatment and PTX + hUC-MSCs treatment mice testis. The mice were treated with PTX or hUC-MSCs, and samples were collected 2 weeks later. **A**: Testicular expression of NRF2 and SIRT1 in control, PTX and PTX + hUC-MSCs treated mice testes; **B**: Expressions of CAT, SOD1, and PRDX6 in mice testes of control, PTX and PTX + hUC-MSCs group were detected by Western blotting, and analyzed by One-Way ANOVA; **C**: Gene expressions of *Cat*, *Sod1*, and *Prdx6*; *p* value less than 0.05 was considered significance; *, *p* < 0.05; **, *p* < 0.01; ***, *p* < 0.001

Oxidative stress was one of the major adverse effects of PTX treatment on mice testis. Immunohistochemical analysis showed that SIRT1 and NRF2 were down-regulated in PTX-treated testis but that hUC-MSCs treatment maintained its basal expression levels (Fig. 9A). We also detected the antioxidant-related indicators CAT, SOD1, and PRDX6 using Western blotting and RT-PCR in mice testes. The results showed that CAT, SOD1, and PRDX6 expression was significantly down-regulated in PTX-treated mice but up-regulated in the PTX + hUC-MSCs group (Fig. 9B). This was consistent with their mRNA expression (Fig. 9C).

### hUC-MSCs affected the expression of apoptosis-related proteins BAX and BCL2

**Fig. 10:**
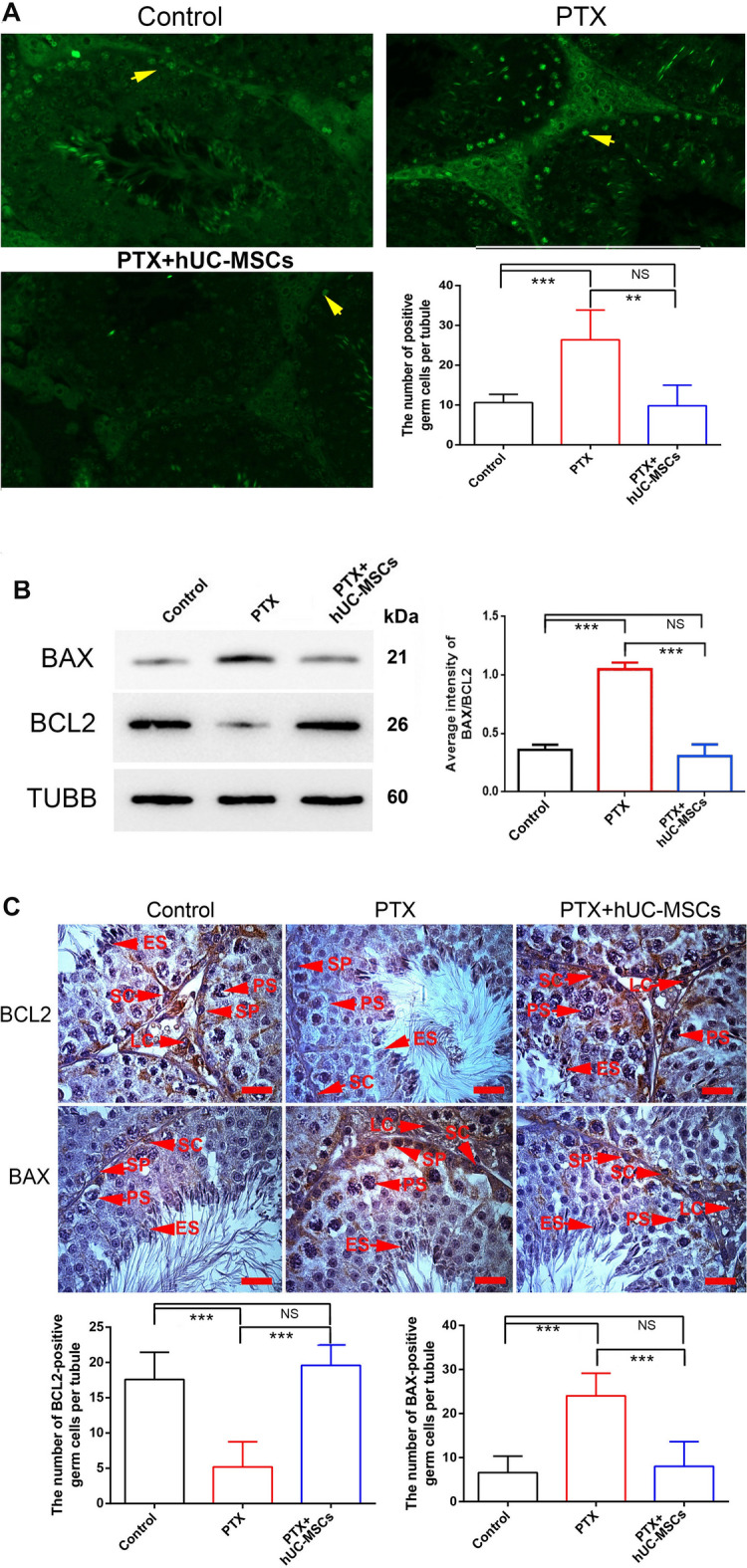
Detection of germ cell apoptosis in control, PTX treatment and PTX + hUC-MSCs treatment mice testis. The mice were treated with PTX or hUC-MSCs, and samples were collected 2 weeks later. **A**: Detection of positive apoptosis germ cells by TUNEL assay, the arrow indicated the apoptosis germ cells; Expressions of BAX and BCL2 in mice testes of control, PTX and PTX + hUC-MSCs group were detected by Western blotting (**B**) and immunohistochemistry (**C**), respectively. Statistical analysis was performed by One-Way ANOVA; *p* value less than 0.05 was considered significance; *, *p* < 0.05; **, *p* < 0.01; ***, *p* < 0.001; Each bar in (**C**) represented 20 μm

Compared with the control group, PTX significantly increased the germ cells apoptosis, while the control group and PTX + hUC-MSCs group showed no significant difference in cell apoptosis (Fig. 10A). PTX treatment also decreased the expression of the anti-apoptosis protein BCL2, and increased the expression of the pro-apoptosis protein BAX. The increased expression ratio of BAX/BCL2 was reversed to normal levels in the PTX + hUC-MSCs group (Fig. 10B). The differential expressions of BCL2 and BAX in the testis were also detected by immunohistochemistry (Fig. 10C).

## Discussion

We previously reported that PTX treatment could affect male mice germ cell proliferation and meiosis, thus affecting sperm quality and male fertility. These changes may be directly related to PTX-induced oxidative stress increases [9]. MSCs play an important role in regulating inflammatory responses, reducing damage from oxidative stress, and regulating immune micro-environments [15, 20]. MSCs have been suggested to play promising roles in repairing in various disease models by their ability to migrate, proliferate and differentiate to repair cells and tissues [21]. Therefore, we hypothesized that hUC-MSCs, the most widely used MSCs, could ameliorate PTX-induced reproductive damage and improve fertility.

In the present study, preliminary experiments were conducted to establish the PTX-treated mice model and determine the appropriate concentration of hUC-MSCs injection. Evidence suggests that hUC-MSCs may exert their effects in the testis, as demonstrated by the detection of labeled hUC-MSCs in the testicular interstitium within 15 min. The stable expression of CD73 is one of the important surface markers to identify hUC-MSCs. As a mouse-derived monoclonal antibody, which was specific for human cytoplasmic marker proteins, STEM121 is usually used in human cell-to-mouse and rat transplantation experiments to monitor cell engraftment, migration, and differentiation. One week later, the expression of CD73 and STEM121 in the testis tissue of the control group (PBS + hUC-MSCs) and PTX + hUC-MSCs treatment group could still be detected. The results suggested that hUC-MSCs can survive for a long time in mouse testis tissue and exert effective paracrine immunomodulatory function to repair tissue damage [22–24]. After 2 weeks, significant changes in testicular morphology and key proteins related to spermatogenesis were identified in PTX-treated mice testis, and the application of hUC-MSCs (2 × 10^6^ cells) significantly improved these changes and elevated antioxidant levels. These findings demonstrated the potential protective effects of hUC-MSCs, which needs a further in-depth investigation.

We also found that the PTX treatment significantly affected mice spermatogenesis. Spermatogenesis in mammals is a cyclic process of spermatogenic cell development in the seminiferous epithelium that can be subdivided into 12 subsequent stages in mice. The stages of VII-VIII are critical for regulating germ cell proliferation. Morphological examination showed that PTX treatment reduced the proportion of stage VII and VIII tubules in the testis, which could also account for the decreased sperm counts in PTX-treated mice. However, after hUC-MSCs treatment, the number of stage VII and VIII tubules increased. Histological analysis also revealed that hUC-MSC administration restored the numbers of germ cells within tubules and improved the loose interstitial structures, which is consistent with the previous reports where hAMSCs restored spermatogenesis in mice with busulfan-induced testis toxicity [12]. Testosterone is believed to directly regulate spermatogenesis [25], and hUC-MSC transplantation increased its levels, which were impaired by PTX treatment. This finding aligns with the results of a study that BMMSCs ameliorated testosterone levels impaired in nitrate-induced rat infertility [26]. Similarly, ADMSCs were reported to restore the testicular size and weight of busulfan-induced azoospermic rats [27], supporting our results.

Abnormal spermatogenesis can directly affect sperm quality, as reflected in the changes in sperm parameters and subsequent male sub-fertility. Sperm counts and progressive sperm motility are crucial diagnostic markers for various infertility conditions and are used to evaluate male fertility potential. The present investigation clearly demonstrated that hUC-MSC treatment significantly increased total sperm numbers and the proportion of progressive sperm motility. Similar effects have been reported in previous studies, where MSCs restored sperm motility in cases of testicular torsion-detorsion injury [28], and BM-MSCs counteracted the detrimental effects of DOX by increasing sperm concentration [29]. Sperm DNA damage had a very clearly positive relationship with low fertilization rates, increased abortion and an elevated incidence of disease in the offspring [30], while it had no relationship with mean embryo score on Day 2 or 3 [31, 32]. Because paternal genome was activated after 2-cell stage, until which point zygotic genome activation took place [33]. Verification of male fertility in vivo indicated that hUC-MSCs treatment recovered normal embryo formation, which reduced significantly in PTX-induced mice. While PTX treatment did not affected two-cell clevage. Combined with these findings of the testis structures, testicular size and weight, testosterone level, sperm parameters, and fertility ability, all of these factors indicated that hUC-MSC administration effectively improves spermatogenesis damage caused by PTX.

Our previous study found that PTX mainly affected spermatogenesis and fertility via impaired germ cell proliferation and meiosis in mice testis. PCNA reflects the proliferation of spermatogonial cells and spermatocytes [34]. hUC-MSCs can significantly improve PTX-caused decreased expression of PCNA in these cells. The result is consistent with a report that Br-MSCs up-regulate PCNA expression of the diabetic islet in type 1 diabetic rats [35]. The meiosis-related molecules SYCP3, MLH1, DMC1, and REC8 were decreased in the testis of PTX-treated mice, but their expression levels significantly improved after hUC-MSCs treatment. Similar to our results, UCB-MSCs increased meiosis-related genes in chemotherapeutic-induced azoospermia mice [36], and SYCP3 expression in busulfan-induced azoospermia could also be restored by UCMSCs transplantation [37]. The fertility-related proteins HSPA2 and HSPA4L were specifically expressed in the testis, and could partially reflect spermatogenesis and the status of sperm quality [38]. In the present study, HSPA2 and HSPA4L were highly expressed in germ cells and down-regulated after PTX treatment, while hUC-MSCs treatment significantly enhanced their expression in germ cells. Adipose-derived mesenchymal stem cells (AD-MSCs) have also been reported to improve sperm quality under H_2_O_2_-induced stress or cryo-damage [39, 40].

Excessive ROS was considered as one of the key reasons for the reproductive damage caused by chemotherapy drugs [41]. Here we found that PTX treatment reduced SOD1, CAT, and PRDX expression in the testis, but they showed significantly increased expression after hUC-MSCs treatment, indicating that hUC-MSCs could enhance testicular antioxidant levels through paracrine or other pathways. We analyzed the expression of NRF2 and SIRT1, the upstream antioxidant regulatory molecules, and found that they had similar expression trends as other antioxidant molecules, suggesting that hUC-MSCs may participate in the regulation of the testicular antioxidant micro-environment via the SIRT1/NRF2-SOD1/CAT/PRDX pathways [42]. Another report also indicated that Nrf2/NQO-1 signaling pathway played an important role in the therapy of non-alcoholic steatohepatitis using hUC-MSCs [43]. AD-MSCs were also reported to be effective in treating psoriasis by negatively regulating ROS [44].

Excessive ROS can promote the apoptosis of testis cells [45, 46]. BAX and BCL2 are key molecules that can reflect cell apoptosis. BAX expression in the PTX group was significantly increased, while BCL2 expression was relatively decreased. hUC- MSCs could reverse both of these expression trends. These results suggest that BAX is one of the key PTX-induced nodes of apoptosis. Additionally, decreased expression of SOD and other antioxidant molecules could also underlie the abnormal expression of BAX and other apoptosis-related molecules, which further affect spermatogenesis and sperm quality, and lead to significant declines in fertility. MSCs’ anti-apoptosis role has also been reported. Human amniotic membrane-derived mesenchymal stem cells (hAMSCs) can ameliorate X-irradiation-induced testicular injury by reducing apoptosis [16]. An important mechanism that the bone marrow-derived mesenchymal stem cells (BM-MSCs) protects against cisplatin-induced gonadotoxicity is anti-apoptosis [47].

Spermatogenesis is an orderly cascade process that is successfully completed via the precise regulation of genes, proteins, and various cytokines [48]. This paper mainly examined changes in male spermatogenesis and sperm fertility induced by PTX, as well as the protective role of hUC-MSCs. The underlying mechanism could be attributed to the antioxidant and anti-apoptosis properties of hUC-MSCs through up-regulating antioxidant markers such as SOD1, CAT, PRDX, Nrf2, and SIRT1, while downregulating apoptosis markers. The protective effects of MSCs are likely associated with their ability to secrete various cytokines which participate in testis development and hormone synthesis, improve spermatogenesis and the sperm maturation micro-environment, and affect sperm quality and male fertility [49]. These results provide an important basis for further detailed molecular mechanism studies.

A single administration of hUC-MSCs could restore spermatogenesis and male fertility potential in the PTX-induced mice model, with antioxidant and anti-apoptosis characteristics being responsible for this effect. This study provides important information for research on male fertility protection. hUC-MSCs may be a promising agent for maintaining sperm quality in chemotherapy treatment.

### Supplementary Information

**Additional file 1: Figure S1.** Examination of spermatogenesis stages and morphology in mice testes from different concentration of hUC-MSCs treatment groups. The mice were treated with hUC-MSCs, and samples were collected one week later. The data were analyzed by one-way ANOVA; *p* value less than 0.05 was considered significance; *, *p*<0.05; **, *p*<0.01; ***, *p*<0.001.

**Additional file 2: Figure S2.** Tracking of injected hUC-MSCs in different mice tissues. Detection of CFDA-SE labelling hUC-MSCs under fluorescence microscope in mice heart, liver, spleen, lung and kidney at different time point. The slides were obtained from frozen section, and green signals show the present of hUC-MSCs.

## Data Availability

Not applicable.
