# Supplementary figures and images for: Human umbilical cord mesenchymal stem cells (hUC-MSCs) alleviate paclitaxel-induced spermatogenesis defects and maintain male fertility
Source: Biol Res. 2023 Aug 13;56:47. doi: 10.1186/s40659-023-00459-w (PMC10424423; doi:10.1186/s40659-023-00459-w)

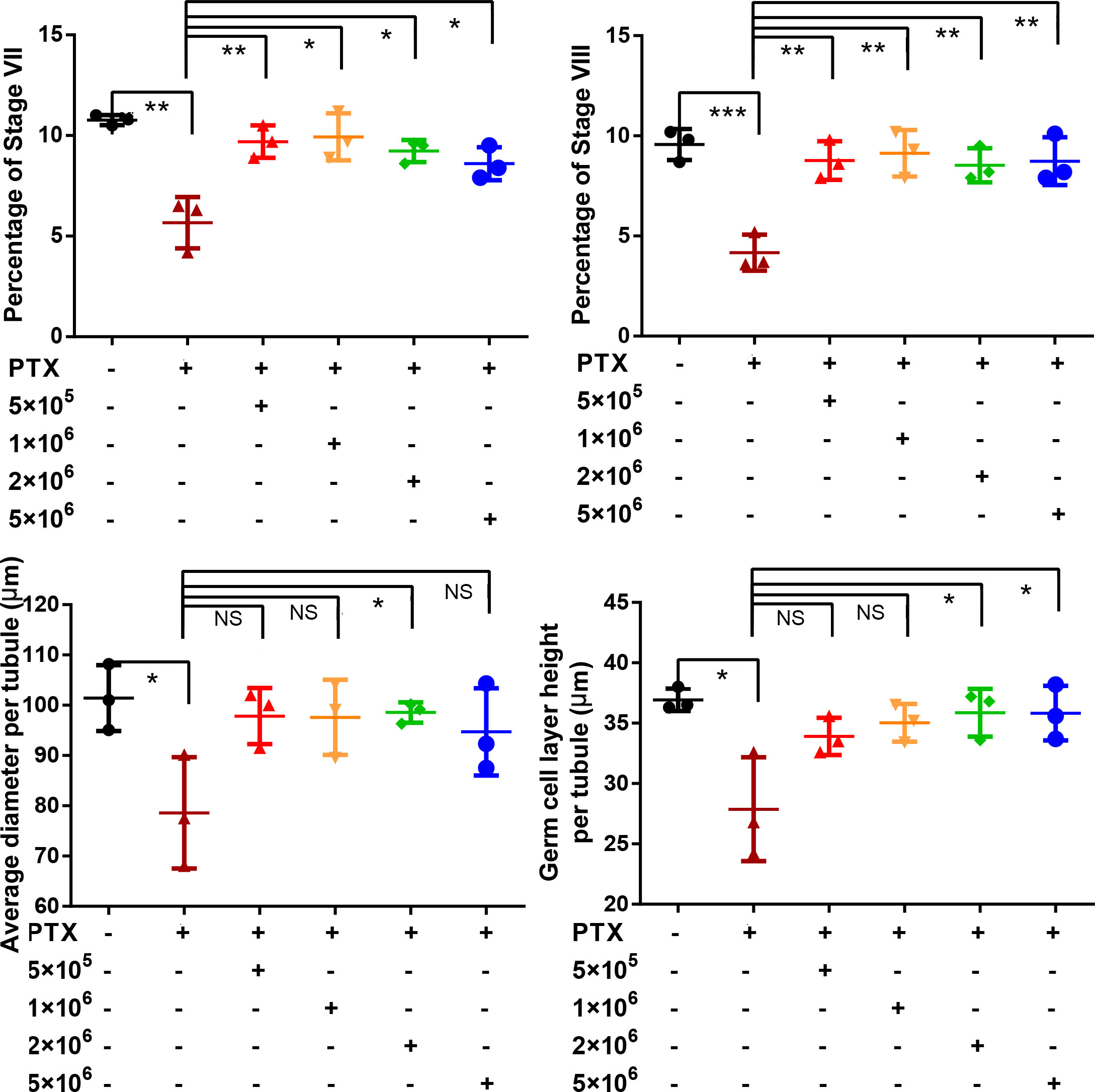

Supplement: Supplementary file 1 — Additional file 1: Figure S1. Examination of spermatogenesis stages and morphology in mice testes from different concentration of hUC-MSCs treatment groups. The mice were treated with hUC-MSCs, and samples were collected one week later. The data were analyzed by one-way ANOVA; p value less than 0.05 was considered significance; *, p<0.05; **, p<0.01; ***, p<0.001. [file 40659_2023_459_MOESM1_ESM.jpg]

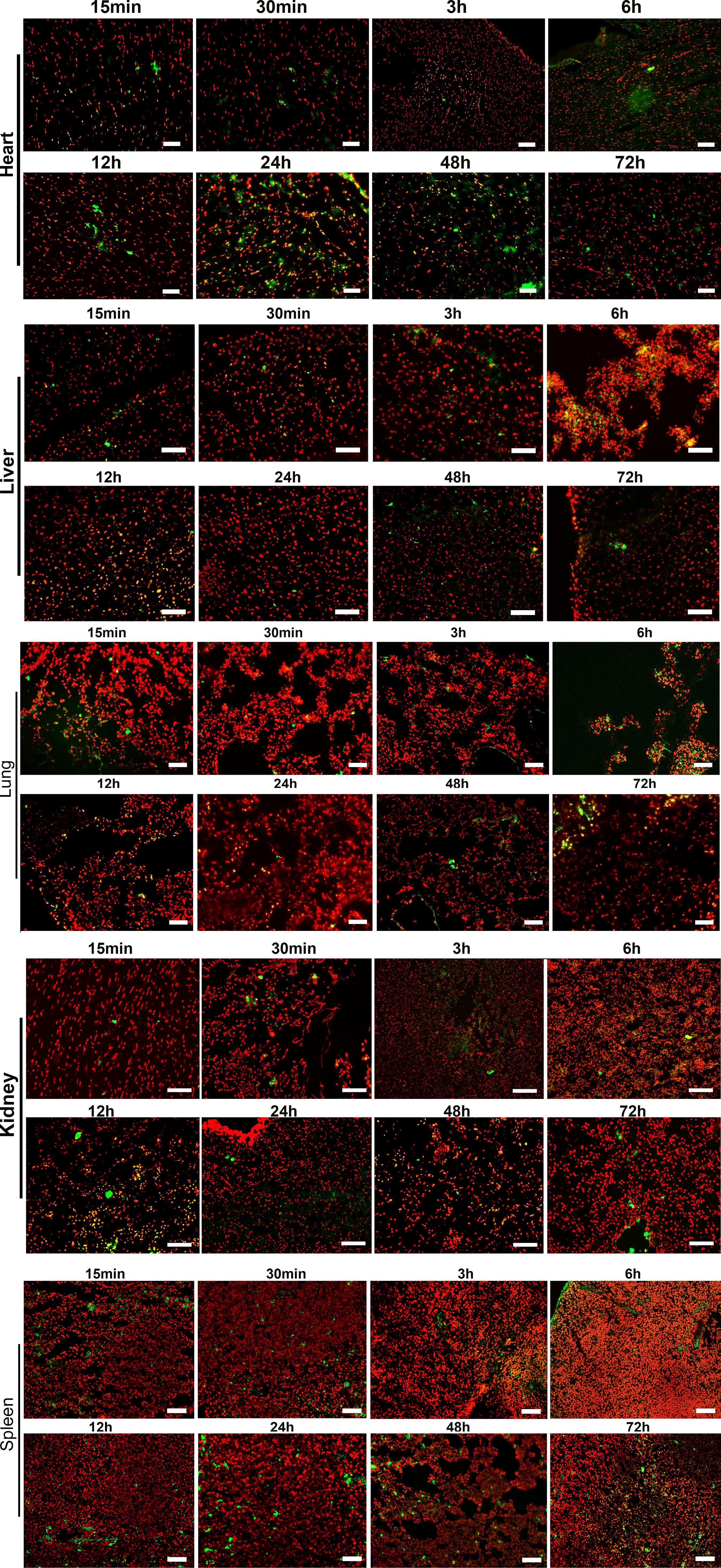

Supplement: Supplementary file 2 — Additional file 2: Figure S2. Tracking of injected hUC-MSCs in different mice tissues. Detection of CFDA-SE labelling hUC-MSCs under fluorescence microscope in mice heart, liver, spleen, lung and kidney at different time point. The slides were obtained from frozen section, and green signals show the present of hUC-MSCs. [file 40659_2023_459_MOESM2_ESM.jpg]
